# Effect of Exchange Dynamics on the NMR Relaxation of Water in Porous Silica

**DOI:** 10.1021/acs.jpclett.4c02590

**Published:** 2024-11-05

**Authors:** Bulat Gizatullin, Carlos Mattea, Siegfried Stapf

**Affiliations:** † Dept. Technische Physik II/Polymerphysik, Technische Universität Ilmenau, D-98684 Ilmenau, Germany

## Abstract

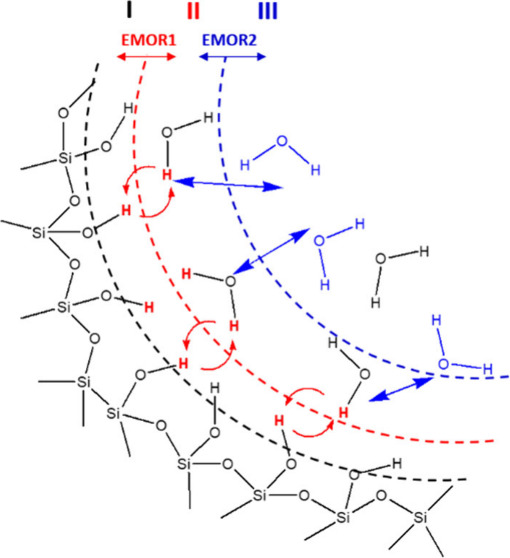

The interaction of molecules, in particular, water, with solid interfaces has been studied by a multitude of methods, among them nuclear magnetic resonance spin relaxation. The frequency dependence of the relaxation times follows patterns that have been interpreted in terms of the molecular orientation and dynamics. Several different model approaches could successfully explain limiting cases of ^1^H relaxation dispersion in systems with rigid surfaces such as silica gel or glass, but none of them can reproduce the relaxation of both ^1^H and ^2^H nuclei, which differ in their respective relaxation mechanisms, dipolar vs quadrupolar. From detailed studies of the dynamics of hydration of water in biological materials, the importance of hydrogen and molecular exchange to the longitudinal relaxation time of T_1_ was demonstrated. In this work, exchange times of both H_2_O and D_2_O in hydrophilic silica gel are varied in a controlled fashion in a wide range using disodium hydrogen phosphate, and the effect of physical exchange on spin relaxation is quantified for the first time in such systems using the exchange-mediated reorientation model.

The study of water dynamics is among the most challenging and puzzling scientific subjects.^1−3^ Many features of physicochemical processes related to the unique properties of water molecules have been investigated with various methods and techniques for decades.^4,5^ The structure of liquid water, hydrogen bonding effects, and solvent properties are only a few of the many fascinating features related to water. Water, the most abundant molecule in living organisms, participates in most of the physical and chemical processes in the living species.^6^ Therefore, many studies and applications, including NMR as a powerful in vivo method, aim to use water as the primary information carrier about the system under study. Understanding the dynamics of water in model systems, such as porous silica, is crucial for further interpretation and application of the role of water in biological systems. The surface properties of the native polar silica surface containing hydroxyl groups can be modified by adsorbing or chemically bonding different substances. This allows variation of the polarity and hydrophobicity as well as interaction types characterizing the adsorbed liquids and silica surface, which also provides access to some natural processes, including biological objects. Most analytical methods were applied with the aim of studying the short time behavior of water, which was found to become extended by 1 or 2 orders of magnitude comparable to its bulk rotation time of a few ps, e.g., by using optical Kerr effect,^7,8^ 2D IR^9−12^, and MD simulations.^13,14^ The latter find water jump times up to 100 ps but are not able to cover longer time scales. Dielectric measurements of water in a porous silica glass showed a similar process plus a step in the kHz–MHz range that was assigned to hydrogen exchange.^15,16^

The frequency dependence of NMR relaxation times, also known as nuclear magnetic relaxation dispersion (NMRD), provides information about molecular motions and interactions modulating local magnetic fields in the wide range of characteristic correlation times of about 10^–9^–10^–4^ s which is covered by the fast field cycling (FFC) technique.^17^ While the time scale of free, or bulk, molecular motions at ambient temperatures on the order of picoseconds is out of the observable range of conventional NMRD times range, processes on the surface of porous materials are much slower, becoming accessible with NMRD and thus being studied throughout the last 30 years.^17−22^

In many dynamics processes affecting NMR relaxation, the Lorentzian spectral density function is considered for the description of observed NMRD, leading to the expression for homonuclear relaxation rate as follows:^22^
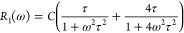
1where τ is a correlation time and ω is the Larmor frequency. The prefactor *C* is proportional to parameters defining interaction in the spin system, such as spin density, distance between interacting spins, dipole–dipole or quadrupolar interaction strength, and electron–nuclear spin–spin interaction. According to eq 1, increasing the rate of dynamics processes, e.g., by increasing the temperature and assuming a constant value of *C*, leads to shifting the NMRD position—the inflection point of the *R*_1_(ω) curve—to higher frequencies. Observing the relaxation rate temperature dependence at constant field, e.g., at 1 T using a conventional benchtop spectrometer, exhibits the characteristic dependence with a maximum at *ωτ* ≈ 0.616. The amplitude of NMRD in the low-frequency limit (*R*_1_(ω → 0)) decreases with an increasing temperature. Using this limit *R*_1_(ω → 0), the value of *R*_2_ relaxation rate can also be estimated.^22^ This, in turn, can be used in applications where the *R*_2_ relaxation rate plays a key role, e.g., in MRI contrast adjustment.

Extracting meaningful dynamic parameters from NMRD data analysis, such as correlation times of molecular motion, strongly relies on the choice of suitable models. In the general case, one may define the total relaxation rate as a sum of intra- and intermolecular relaxation contributions which are further subdivided by a number of specific components as follows:
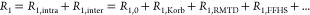
2where *R*_1,Korb_ is paramagnetic relaxation related to the Korb model,^26^*R*_1,RMTD_ refers to the reorientations mediated by translational displacements (RMTD) model,^19^ and *R*_1,FFHS_ is the force-free hard sphere (FFHS) model contribution^23,24^ (see below). *R*_1,0_ then describes the behavior of the bulk liquid, which is a constant within the NMRD frequency window and is fully explained by rotational and diffusional terms. The variety of dynamics models^32,33^ and the complexity of the system under study require a significant amount of supplementary data, such as diffusion coefficients, electron relaxation times, internuclear distances, or alternatively some simplifying assumptions, e.g., one contribution dominating over the other, idealization of the molecular structure, *etc.*

All of the mentioned contributions calculate the influence of molecular motion in the vicinity of a surface and are shown to result in NMRD curves that are compatible with experimental results in limiting situations. For instance, surface diffusion along a material with paramagnetic impurities at the surface or within the bulk solid^26−28^ assumes that electron–nuclear spin–spin interaction dominates over other effects. As is often the case in porous glass and silica in general and was specifically verified in this study, the concentration of paramagnetic centers is too low (below nmol/L as determined by EPR spectroscopy) for this contribution to become significant. However, attempts to apply this Korb model of effectively two-dimensional surface diffusion to the system without paramagnetic centers have been reported.^34^ In an earlier study on liquids in natural rocks where the Korb model was expected to apply for ^1^H, we were not able to confirm the expected scaling behavior when employing ^2^H homologues; in fact, the NMRD shapes changed fundamentally.^29^

Models that appear more suitable for describing the present system are related to the RMTD and FFHS concepts, which are considered as intra- and intermolecular contributions to the relaxation, respectively. The RMTD relaxation mechanism is entirely based on reorientations of molecules due to their consecutive adsorption and desorption processes, the surface orientations assumed to be a consequence of unspecified interaction. Following such adsorption/desorption processes, the molecule possesses “memory” about its orientation relative to the surface and to the static external magnetic field, sampling information about the surface structure, curvature, and roughness during its diffusion. In the FFHS model, molecules are considered as hard spheres carrying spins at their centers. The molecules experience three-dimensional isotropic translational diffusion and elastically collide with each other. The other interactions between molecules are neglected.

Application of one of the mentioned models to liquids on surfaces often delivers acceptable fits with characteristic correlation times that are difficult to verify. One possible experimental approach to distinguish or emphasize specific dynamics contributions to the NMR relaxation is therefore the systematic variation of controllable parameters of the systems, which include surface modifications,^35^ polarity of adsorbates,^29^ temperature and pH,^36^ and discussing their expected consequence within the specific model framework. For instance, as a successful approach, the comparison of ^1^H of NMRD of an adsorbed liquid and ^2^H NMRD of its deuterated analogue in a particular macroporous system was demonstrated in refs (10, 37, and 38) where the equivalence of the NMRD shape between both nuclei suggested that the RMTD mechanism is applicable. However, we have found that this is not observed in general and that both nuclei feature different NMRD shapes in the majority of porous materials. Therefore, in our previous contributions,^29−31^ a new approach applying the subtraction of normalized ^1^H and ^2^H NMRD was suggested to separate the intra- and intermolecular contributions to the NMR relaxation. One of the remarkable effects requiring deeper analysis was the presence of a significant intermolecular contribution to the NMRD of water in fumed silica and SBA-15 material.^35^ Additionally, the modification of the silica surface with NH_2_ groups led to an increasing ^2^H NMRD relaxation rate at the low-frequency limit of up to 1 order of magnitude, which was tentatively assigned to the proton exchange between NH_2_ groups and water molecules. By comparison with any of the mentioned relaxation models, the change of the correlation times of the corresponding mechanism was in the expected range while the NMRD amplitudes ^1^H and ^2^H were not, making the reasonable assumption of very similar dynamic time scales for H_2_O and D_2_O molecules. In line with the literature discussing the mentioned models of surface diffusion (Korb), RMTD ,and FFHS, the possible influence of physical exchange with surface hydroxyl groups, present at a rather high surface density of up to 5–6 groups per nm^2^ in untreated silica,^39^ was not taken into account based on the assumption of a slow exchange rate, which should render it negligible compared to the contribution of other reorientation mechanisms.^40−43^

Thus, the question of adequately addressing the exchange contribution to ^1^H and ^2^H NMRD after silica surface modification remained open. In the current contribution, we present one step further in understanding the exchange effect in the studied systems by enabling exchange rate variation with the presence of an exchange catalyst. Furthermore, an additional motivation for this study was the reproducibility of the NMRD curves and the correctness of the corresponding analysis when species such as isotopes or stable organic radicals are added to the systems as labels in an amount usually counted as negligible to alter the dynamics in the systems significantly. In those cases, the additional relaxation rate due to the presence of paramagnetic species is conventionally counted as linearly scaled with their concentration. However, the amplitude or shape of either ^1^H or ^2^H NMRD may be additionally affected by the altered exchange rate due to any exchange catalysts in the system. This effect can also be an indirect result of surface modification due to the presence of residual chemicals or buffers. The latter can perform as an exchange catalyst, which was controlled in the current contribution using phosphate salt. Thus, distinguishing and understanding the exchange contribution to the NMR relaxation becomes even more crucial for further development of dynamics models and numerical analysis of NMRD data.

In the current contribution, we present the results of a study of the exchange effect on ^1^H and ^2^H NMR relaxation dispersion of water in fumed silica employing, for the first time, the exchange-mediated orientational randomizations (EMOR) model originally suggested for biopolymers.^44−46^ The hydrogen or deuteron exchange rate was controlled by the presence of an exchange catalyst, namely, disodium phosphate, in the range of concentrations of 0.5–32 g/L, equivalent to 3.5–225 mmol/L. The analysis of both ^1^H and ^2^H NMRD was carried out using an analytical approximation of the EMOR model with the same correlation times related to exchange, which were used as a shared parameter for each phosphate concentration in a global fitting procedure of both ^1^H and ^2^H NMRD simultaneously. The difference in exchange times as a consequence of the isotope effect was considered negligible at this stage. We have found evidence of two dynamic components; these are discussed in terms of hydrogen–deuteron exchange between labile protons of water molecules and hydroxyl groups on the surface as well as adsorption strength in the system.

Relaxation dispersion measurements were carried out by using an FFC relaxometer (Spinmaster FFC2000, Stelar, Mede, Italy) at magnetic field strengths between 0.1 mT and 0.6 T. For the NMR signal acquisition, the probes were tuned to 16.7 or 3.4 MHz for ^1^H and ^2^H target nuclei, respectively. These values were optimized to guarantee the best signal-to-noise ratio while keeping instabilities due to magnet heating to a minimum; the choice of the acquisition field has no effect on the measured relaxation times.

Before adding either disodium hydrogen phosphate (>99% of purity, Carl Roth GmbH, CAS 7558-79-4) solution in D_2_O (99.9% of isotopic purity, Deutero GmbH, Germany) or bidistilled H_2_O to the fumed silica powder with 7 nm particle size and 380 m^2^/g surface area (Sigma-Aldrich, CAS 112945-52-5), samples were dried in a vacuum oven for 24 h at 390 K and less than 10 mbar in order to remove residual moisture from silica powder. The pure D_2_O/H_2_O and corresponding Na_2_HPO_4_ solutions were added to the silica powder at a 2 mL/g concentration. The measurements were performed after 48 h. All measurements were carried out at 293 K.

The experimental ^2^H and ^1^H NMRD results of pure D_2_O and H_2_O and the corresponding Na_2_HPO_4_ solutions at different concentrations in fumed silica are presented in Figure 1.

**Figure 1:**
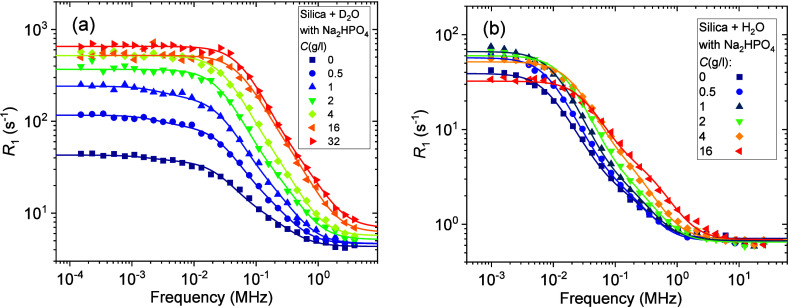
^2^H (a) and ^1^H (b) NMRD of D_2_O and H_2_O, respectively, at different concentrations of Na_2_HPO_4_ in fumed silica. The lines are fittings using eqs 3 and 5–8.

Considering the full ^2^H NMRD data set, the overall shape of all NMRD curves can be regarded as similar. A detailed analysis of the NMRD normalized to the low-frequency limit amplitude (see Figure S2) reveals that the shape remains virtually unchanged up to a disodium phosphate concentration of 2 g/L; above that value a shift of the dispersion toward higher frequencies occurs. Above 16 g/L Na_2_HPO_4_, the NMRD spectra remain consistent in amplitude and position. The shift of the curves toward higher frequencies is a clear indication of the dynamics rate increase; that is, molecular motions are becoming faster. However, the models mentioned in the introduction, e.g., isotropic continuous rotational diffusion^30^ or reorientation mediated by translational displacements,^30,38^ all predict a decrease in the NMRD amplitude when the correlation times decrease. The hypothesis about exchange effects on NMRD brings the two-site exchange model according to Zimmerman and Brittin^47^ into consideration. Although that model can explain tendencies in amplitude change due to temperature variation, the reason for the dispersion position behavior, including the shift to higher frequencies after adding an exchange catalyst, is obscure in terms of that model. The temperature change, even in a narrow range, exhibits similar unusual changes in NMRD amplitude, i.e., increasing relaxation rate with increase of temperature, while the value of *R*_1_ at high frequencies decreases (see Figure S2).

The exchange-mediated orientational randomization (EMOR) model introduced by B. Halle and coauthors^44−46,48,49^ is a well-described concept of spin dynamics derived using the stochastic Liouville equation (SLE) that has been applied in only a few studies^36,46,50^ mostly related to biological systems, but not yet to solid porous media such as porous silica. The idea of the EMOR model is based on the concept of exchange, which occurs when internal water molecules or labile hydrogens escape from orientationally confining sites. The main difference between the EMOR model and the other models that consider exchange is that EMOR not only mixes the states but induces relaxation by itself. This means the direct presence of a contribution to the NMRD related to exchange–correlation times. Thus, we consider our system a porous medium presented by fumed silica with different pools of spins belonging to adsorbed liquid and participating in the exchange processes (see Figure 2).

**Figure 2:**
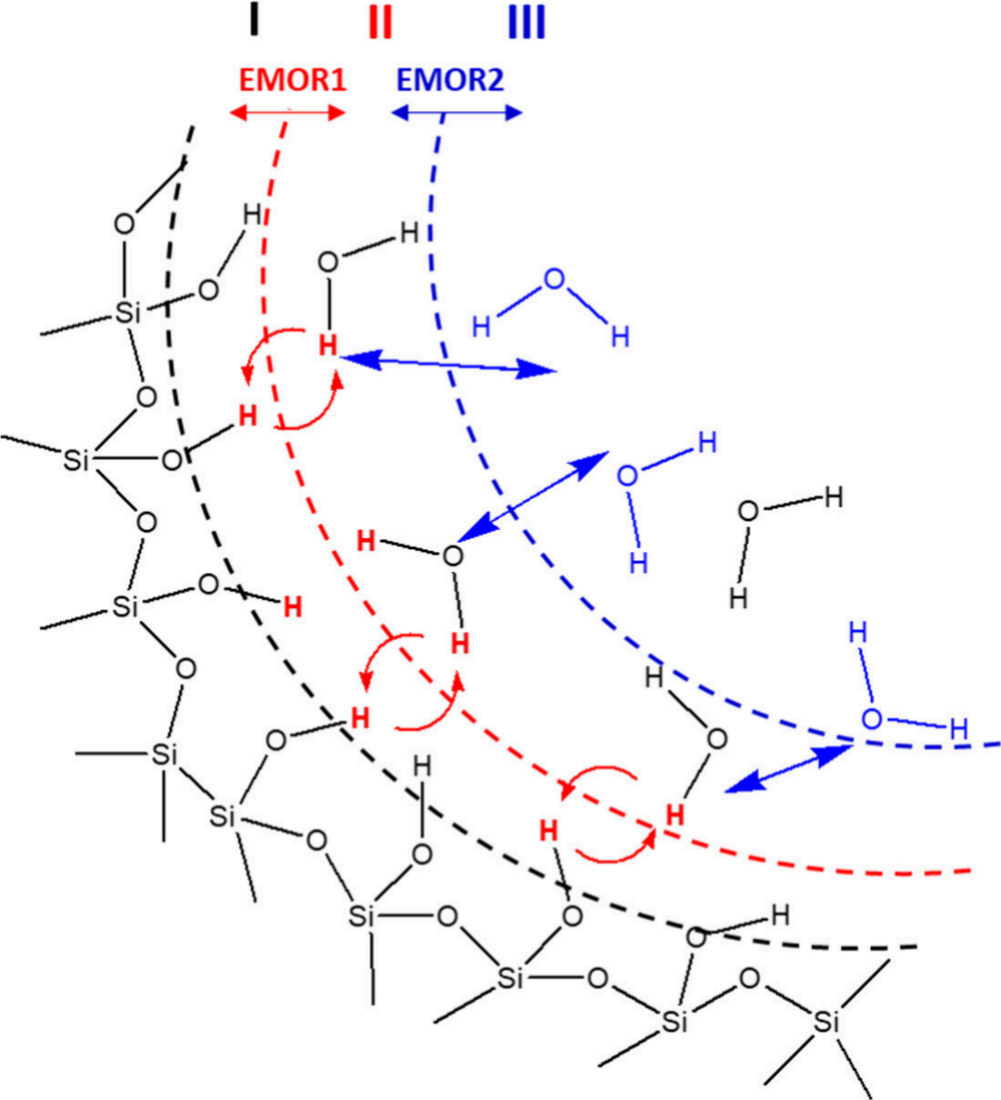
Exchange model of adsorbed water molecules on the silica surface includes hydroxyl groups on the silica surface (I), adsorbed water molecules (II), and bulk water (III) with respective exchange processes “EMOR1” and “EMOR2” described by the EMOR model (see eqs 3 and 5).

The first process is viewed as the exchange of labile hydrogens or deuterons between the adsorbed water molecules and hydroxyl groups on the silica surface. The correlation times of exchange of labile hydrogens or deuterons in water systems, e.g., with proteins or other biological tissues, are of a time scale above 1 ms^36,40,42,45,51,52^ depending on the pH of the systems and origin of labile hydrogen, e.g., in amino, carboxyl, or hydroxyl groups, as well as the presence of exchange catalysts.^40^ The second process is related to the ubiquitous physical exchange between adsorbed water molecules and bulk water molecules that are distanced from the silica surface. Furthermore, in the EMOR model, the exchange not only mixes the spin populations but also directly modulates spin relaxation in the system. Considering ^2^H relaxation, the relaxation rate of the first exchange process *R*_1,EMOR1_ mentioned above is defined as^45^
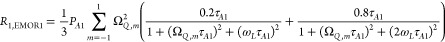
3where *P*_*A*1_ is a population in the anisotropic state where the nuclei are subject, in addition to the Zeeman coupling, to a residual quadrupole coupling with quadrupole frequency ω_*Q*,*i*_ = *S*_*i*_ω_*Q*_^0^ with ω_*Q*_^0^ = 3*πχ*/2. *S*_*i*_ is the order parameter for the *i*th anisotropic phase, which describes the incomplete motional averaging of the quadrupole frequency related to the quadrupole coupling constant χ (in Hz). τ_*A*1_ is the exchange time, and ω_*L*_ is the nuclear Larmor frequency; the NQR frequencies Ω_*Q*,*m*_ = *c*_*m*_ω_*Q*1_ and coefficients *c*_*m*_ are related to differences between eigenvalues of the quadrupole Hamiltonian as^45^
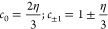
where η is the electric field gradient (EFG) asymmetry parameter.

Equation 3 is derived as an analytical approximation of the SLE approach used in the original papers^45,46^ related to dilute systems in a motional-narrowing regime. When an ultraslow motion regime is achieved, i.e., Ω_*Q*,*m*_τ_*A*1_ ≫ 1, and assuming an axially symmetric EFG tensor, i.e., η = 0, the relaxation rate is expressed as^45^
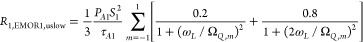
4Thus, when the exchange is slowed down and τ_*A*1_ becomes longer, the contribution to the relaxation related to the EMOR model is revealed as the NMRD is no longer shifted to lower frequencies but instead is decreasing in amplitude with increasing τ_*A*1_: *R*_1,EMOR_(ω → 0) ∝ 1/τ_*A*1_, opposite to the tendency *R*_1_(ω → 0) ∝ τ corresponding to eq 1. Thus, the information about exchange correlation times is encoded not only in the shape but also in the amplitude of the NMRD. In addition, the signature of the presence of exchange, which can be described with the EMOR model, explains the increasing NMRD amplitude when the temperature is increased while the position remains invariant (see Figure S2).^45^ It should be emphasized that the latter effect is observed for water in silica also in the absence of phosphate salt, demonstrating the necessity to always consider the EMOR model contributing to the NMR relaxation in similar systems, even in the absence of an exchange catalyst.

Furthermore, in the motional-narrowing limit, a second exchange process is assigned to physical exchange between adsorbed and bulk water molecules, which takes place on a submicrosecond time scale^21,29,31,49,52,53^ and is characterized by the following equations:^45,55^
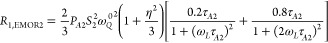
5where *P*_*A*2_ is the population in the anisotropic state subjected to the molecular exchange between water molecules adsorbed on the surface and bulk with exchange time τ_*A*2_ that is assigned to the motional-narrowing limit.

Employing the same assumptions and limits to analyze ^1^H NMRD of water (see Figure 1b), it is possible to carry out a fitting procedure using equations equivalent to eqs 3 and 5 assuming like-spin relaxation with ω_*Q*_ replaced by the dipolar frequency ω_*Di*_ = *S*_*i*_ω_*D*_^0^ = *S*_*i*_3χ_*D*_/2 with the dipole coupling constant χ_*D*_ (in rad s^–1^) given by χ_*D*_ = μ_0_γ^2^*ℏ*/4*πr*^3^, where *r* is the interspin distance. Thus, the experimental ^1^H NMRD in Figure 1 are fitted using the following equations:^45,46^
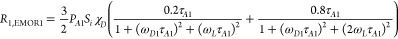
6
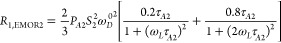
7The fitting of experimental NMRD, acquired for both ^1^H and ^2^H on either H_2_O or D_2_O samples, was carried out using τ_*A*1_ and τ_*A*2_ as shared parameters in a global fitting procedure. Here it has been assumed that all physical parameters, in particular, correlation times, between water molecules containing ^1^H and ^2^H are almost identical. The quadrupole and dipolar frequencies ω_*Q*_^0^ = 8.7 × 10^5^ rad s^–1^ and ω_*D*_^0^ = 1.5 × 10^5^ rad s^–1^,^45^ respectively, and the asymmetry parameter for quadrupolar interaction η = 0.11^56^ were fixed in the fitting procedure. The results of the fitting are presented in Table 1. Finally, if one does characterize the system by two pools of water molecules and hydroxyl groups on the surface undergoing the exchange processes (see Figure 2) with substantially different exchange rates, the total relaxation rate can be expressed as^45,46^
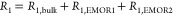
8where *R*_1,bulk_ is the relaxation rate related to the internal motion of the water molecules in the bulk state, which is usually characterized by a picosecond time scale and therefore a constant value within the observed Larmor frequency range. *R*_1,EMOR1_ and *R*_1,EMOR2_ are expressed by eqs 3 and 5 for ^2^H nuclei, and eqs 6 and 7 for ^1^H nuclei, respectively.

**Table 1:** Results of the EMOR Model Fit to ^1^H and ^2^H NMRD of Water in Fumed Silica at Different Na_2_HPO_4_ Concentrations (see Figure 1) Using Equations 3 and 5–8

			*P*_A1_, ×10^–3^		*P*_A2_S_2_^2^, ×10^–3^		*S*_1_		*R*_1,bulk_, s^–1^	
*C*, g/l	τ_*A*1_, μs	τ_*A*2_, ns	^1^H	^2^H	^1^H	^2^H	^1^H	^2^H	^1^H	^2^H
0	139 ± 4	424 ± 45	7.3 ± 0.2	4.8 ± 0.2	0.29 ± 0.02	0.024 ± 0.004	0.87 ± 0.05	0.50 ± 0.06	0.71 ± 0.02	4.4 ± 0.1
0.5	83 ± 2	423 ± 42	6.5 ± 0.1	8.8 ± 0.3	0.31 ± 0.02	0.048 ± 0.006	0.86 ± 0.04	0.40 ± 0.03	0.66 ± 0.01	4.7 ± 0.1
1	23 ± 0.7	417 ± 47	2.4 ± 0.2	6.4 ± 0.3	0.37 ± 0.02	0.09 ± 0.009	0.85 ± 0.04	0.33 ± 0.02	0.68 ± 0.02	4.7 ± 0.2
2	6.8 ± 0.6	295 ± 38	1.8 ± 0.5	4.7 ± 0.3	0.4 ± 0.03	0.14 ± 0.01	0.83 ± 0.02	0.34 ± 0.02	0.65 ± 0.02	5.2 ± 0.2
4	4.4 ± 0.3	338 ± 38	1.4 ± 0.1	4.8 ± 0.3	0.70 ± 0.04	0.20 ± 0.02	0.82 ± 0.06	0.36 ± 0.03	0.69 ± 0.02	5.7 ± 0.2
16	2.7 ± 0.3	193 ± 27	1.6 ± 0.1	4.2 ± 0.3	1.0 ± 0.1	0.31 ± 0.02	0.82 ± 0.05	0.45 ± 0.01	0.68 ± 0.02	6.3 ± 0.5

As seen in Table 1, the exchange time τ_*A*2_ remains essentially independent of Na_2_HPO_4_ concentration, while the exchange time τ_*A*1_ (see eq 3) strongly depends on concentration of disodium phosphate, exhibiting approximately a 2 orders of magnitude change in the range of 0–16 g/L concentration of Na_2_HPO_4_. Similar behavior of the exchange rate in the system with labile hydrogen, e.g., amino acid solutions,^40^ was reported as a result of the exchange rate increase due to the exchange catalysis via phosphate species. The EMOR component which is assigned to the adsorption rate of water molecules on the silica surface exhibits an average τ_*A*2_ of approximately 300 ns independent of Na_2_HPO_4_ concentration. The values of that adsorption time, or residence time, usually obtained applying very well-known models such as RMTD,^19,22^ models involving paramagnetic centers on the surface,^25,26^ and others,^25,31,34,50,53,54^ for materials where these approaches are considered to be valid, are found in the range of 100 ns to 1 μs, in good agreement with our results.

In conclusion, we point out that both ^1^H (dipolar) and ^2^H (quadrupolar) spin relaxation in water molecules in fumed silica, which is usually considered as a suitable porous material to test and display new approaches in dynamics model development and NMRD analysis, can be described with good accuracy with the EMOR model. This model should be considered when the concentration of labile protons in the heterogeneous system, e.g., in hydroxyl or amino groups, is above approximately 1 mM, even in the absence of any hydrogen exchange catalyst, such as phosphate, used in the current work. We would like to emphasize that in most biological systems, where phosphate or other possible exchange catalysts are present, the EMOR mechanism must be considered, without eventually excluding other mechanisms of relaxation related to molecule–surface interaction.

In the current system under study, we present evidence of two exchange processes that define the relaxation reflected in both ^1^H and ^2^H NRMD of water in silica. The validity of the findings can possibly be further verified by employing ^17^O relaxometry which has been discussed before in the context of EMOR of water in proteins.^57^ This approach is only valid for molecular exchange, excluding ^1^H and ^2^H exchange effects on NMRD, giving a clear distinction on which exchange process is addressed. The use of disodium phosphate as a labile hydrogen exchange catalyst allows variation of the hydrogen exchange rate, which is considered the first EMOR contribution, over 2 orders of magnitude and serves as a crucial parameter to prove the validity of the EMOR model. The second EMOR contribution with a characteristic exchange time of about 300 ns independent of Na_2_HPO_4_ concentration is attributed to the readsorption processes of water molecules on the polar silica surface. This readsorption process is considered by many other dynamics models and exhibits an NMRD pattern explained by other models as well.

The concentration and temperature dependence of the amplitude, i.e., the absolute value of relaxation at very low fields, and of the midpoint of the relaxation dispersion as a function of Larmor frequency for water molecules in fumed silica are unconventional and not in agreement with approaches such as Lorentzian spectral density functions or other model predictions in the literature that do not explicitly include exchange. These observations, however, are successfully described by the dominating EMOR contribution in the intermediate and ultraslow regimes. Currently, we apply the EMOR model using analytical approximations valid in the studied system. However, as the authors of the EMOR model insisted on using a rigorous numerical SLE approach for NMRD calculation to reveal all features and effects related to exchange in the system, we will perceive a more detailed analysis in similar systems, including the use of other exchange catalysts as well as modifying the surface to alter and control surface properties and the rate of exchange processes.

## Supporting Information Available

Normalized NMRD, NMRD at different temperatures (PDF)
