# Supplementary material for: Effect of Exchange Dynamics on the NMR Relaxation of Water in Porous Silica
Source: J Phys Chem Lett. 2024 Nov 5;15(45):11335–41. doi: 10.1021/acs.jpclett.4c02590 (PMC11571216; doi:10.1021/acs.jpclett.4c02590)
Supplement: Supplementary file 1 — jz4c02590_si_001.pdf [file jz4c02590_si_001.pdf]

# Supporting Information

## Effect of exchange dynamics on the NMR relaxation of water in porous silica

Bulat Gizatullin, Carlos Mattea, Siegfried Stapf

[1]Dept. Technische Physik II/Polymerphysik, Technische Universität Ilmenau, D-98684 Ilmenau, Germany

Corresponding author: Siegfried.stapf@tu-ilmenau.de, Tel. +49 3677 693671

In the following Figure S 1, the data presented in Figure 1 are processed to set the relaxation rate at the lowest frequency to unity for all measurements. For that purpose, data from fitting curves was used to diminish the effect of scattering of experimental data points. The idea was to show how the NMRD is shifted towards higher frequencies by adding the $Na_2HPO_4$ to the water in silica. The actual shift starts at concentrations higher than 2 g/l. Below that concentration of disodium phosphate, the position of NMRD stays unaltered, showing so-called "saturation" in the original paper about EMOR model of NMRD. This phenomenon is observed in both $^1H$ and $^2H$ NMRD of water in silica.

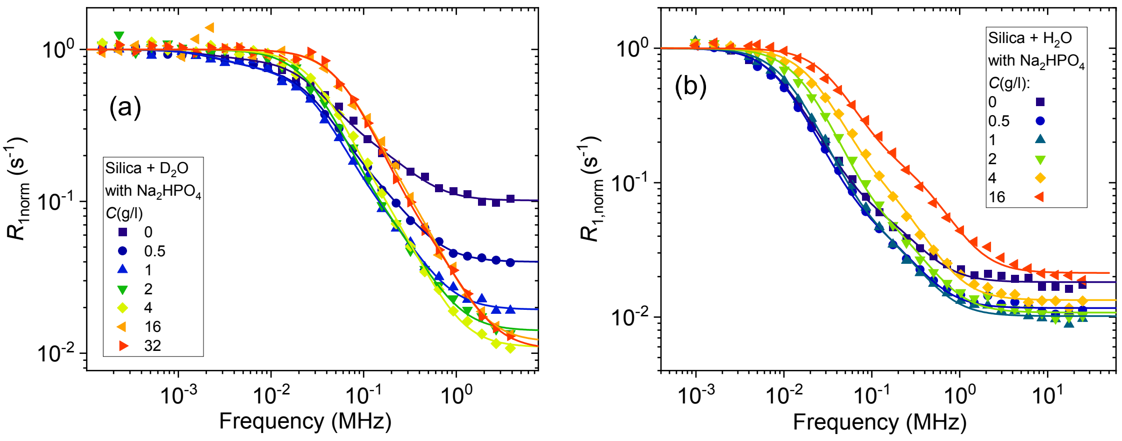


*Figure S 1. Normalized $^2H$ NMRD of $D_2O$ in silica at different $Na_2HPO_4$ concentrations.*

Figure S 2 presents $^2H$ NMRD of $D_2O$ in silica at different temperatures in the range 20-50 °C. Here, the unusual behaviour of NMRD amplitude is presented, i.e., increasing the relaxation rate with the increase in temperature, while generally, the theory predicts the opposite. A similar behaviour with temperature increase is observed for $H_2O$ in silica, too.

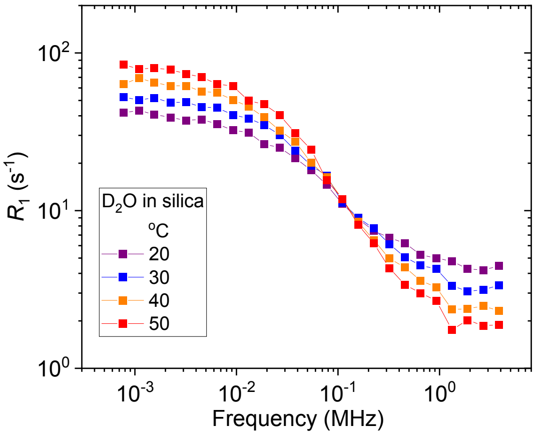


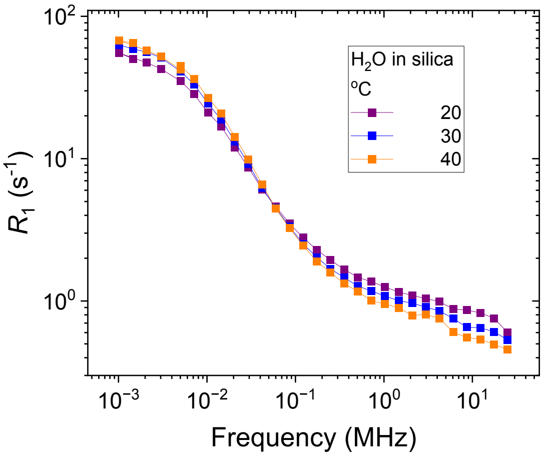


*Figure S 2. $^2$H NMRD of $D_2O$ in fumed silica at different temperatures*
